# Emerging In Vivo Imaging Modalities for Improved Glioblastoma Surgery and Monitoring

**DOI:** 10.3390/biomedicines14040816

**Published:** 2026-04-02

**Authors:** Oluwagbenga Dada, Shikshita Singh, Francheska Sumadchat, Madison Lather, Benjamin Brooks, JuliAnne E. Allgood

**Affiliations:** Rocky Vista University, College of Osteopathic Medicine, Ivins, UT 84738, USA; oluwagbenga.dada@ut.rvu.edu (O.D.); shikshita.singh@ut.rvu.edu (S.S.); francheska.sumadchat@mt.rvu.edu (F.S.);

**Keywords:** glioblastoma, in vivo imaging, single cell resolution

## Abstract

Glioblastoma (GBM) remains the most aggressive primary malignant brain tumor in adults, with poor survival largely driven by diffuse cellular infiltration, profound heterogeneity, and near-universal recurrence following standard therapy. Although maximizing the extent of resection is a key determinant of patient outcome, current clinical imaging modalities lack the spatial resolution necessary to detect microscopic tumor invasion and therapy-resistant cell populations. Emerging in vivo imaging technologies capable of cellular and near-single-cell resolution have therefore become a major focus in preclinical neuro-oncology research, with growing relevance for surgical guidance, treatment adaptation, and translational discovery. This review evaluates multiple optical imaging modalities, including multi-photon microscopy, near-infrared II fluorescence imaging, bioluminescence imaging, photoacoustic imaging, optical coherence tomography, confocal laser endomicroscopy, Raman spectroscopy, autofluorescence microscopy, and fluorescence macroscopy with a focus on their ability to detect residual GBM cells. Despite significant advances, these approaches remain constrained by limitations in molecular target availability, probe delivery across the blood–brain barrier, and signal variability within heterogeneous tumor regions. The biological complexity of GBM further challenges detection, as residual tumor cells are spatially dispersed and phenotypically diverse, limiting the effectiveness of single-marker or single-modality strategies. Together, these findings highlight the need for integrated, biologically informed imaging approaches to improve detection of residual disease and guide surgical decision making.

## 1. Introduction

Glioblastoma (GBM) is the most common and lethal primary malignant brain tumor in adults [1]. It is characterized by profound intratumoral heterogeneity, diffuse infiltration, rapid proliferation, and near-universal recurrence [2,3]. Despite maximal safe resection followed by radiotherapy and temozolomide, median survival remains 12 to 16 months, and fewer than 10% percent of patients survive beyond five years [4,5,6]. Mortality is driven largely by tumor recurrence, which arises from infiltrative glioma cells that evade surgical resection and remain undetectable by standard postoperative imaging [7,8]. Even a small population of residual tumor cells is sufficient to initiate regrowth, underscoring the critical need to identify and characterize all malignant cells within the tumor field.

A defining feature of GBM is the speed and subtlety of its invasion. Preclinical luciferase-based imaging studies demonstrate that glioma cell infiltration occurs early in tumor development, with diffuse integration into surrounding brain tissue well before a discrete mass becomes visible on MRI [9,10]. Consistent with this behavior, contemporary studies indicate that more than 78% of recurrences arise within 2–3 cm of the resection cavity, reflecting the persistence of microscopic disease beyond radiographically defined margins [11]. These infiltrating populations include glioma stem like cells (GSCs), which possess self-renewal capacity, quiescence, lineage plasticity, and enhanced DNA repair mechanisms that promote survival following chemoradiation and drive long-term recurrence [12,13,14]. Surviving tumor cells further acquire therapy tolerance through chromatin remodeling, metabolic reprogramming, and immune evasion, rendering subsequent treatments increasingly ineffective [15].

Conventional imaging plays a central role in GBM diagnosis and surgical planning, particularly in defining tumor boundaries and guiding maximal safe resection. High-resolution, accurate imaging is essential to understand the tumor field, and define the surgical approach and the extent of resection (EOR). However, current clinical imaging modalities are fundamentally limited to tissue-scale resolution and cannot reliably detect the dispersed infiltrative cells responsible for recurrence. This resolution gap represents a major barrier to improving surgical precision and long-term disease control.

A critical unmet need in GBM management is therefore the ability to detect, track, and characterize individual infiltrating tumor cells within living brain tissue. Several emerging in vivo imaging technologies capable of cellular or near-single-cell resolution are now under preclinical development. While many of these approaches will remain confined to experimental models, others show genuine promise for clinical translation. This review surveys the major high-resolution in vivo imaging platforms used in GBM research, challenges associated with fluorescent markers in the brain, and the current progress and challenges associated with their translational application.

## 2. Current Landscape of Imaging in GBM

**Figure 1:**
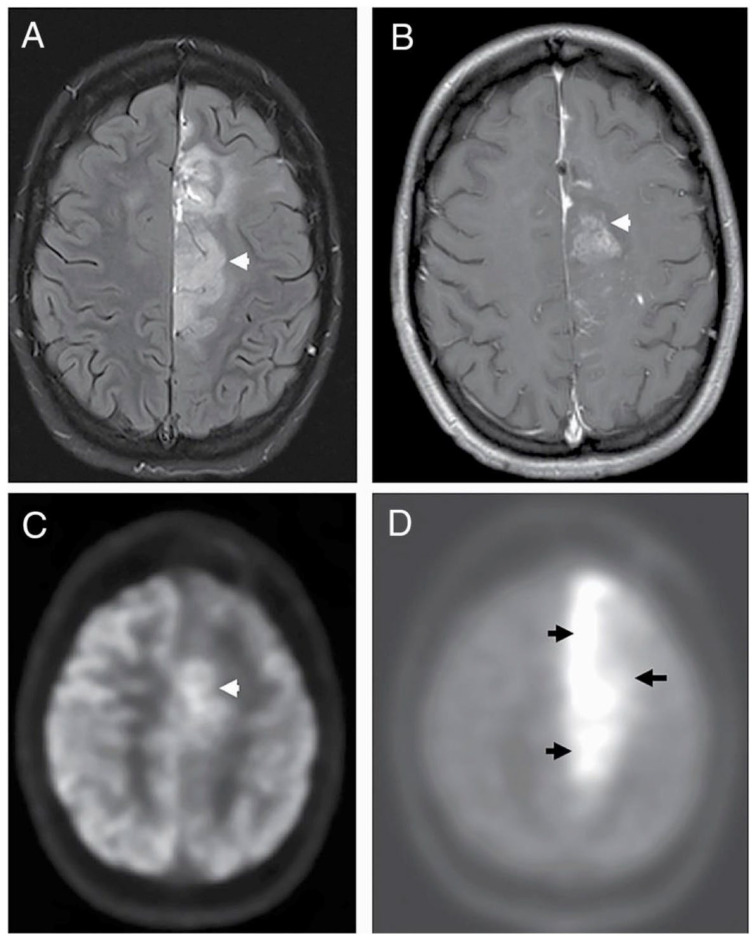
Metabolic imaging of glioblastoma at recurrence. (**A**) Axial T2 FLAIR images through the level of the centrum semiovale demonstrate a large, ill-defined, heterogenous tumor involving the body of the left cingulate gyrus that demonstrates patchy enhancement on the post-contrast T1-weighted sequence. (arrowhead) (**B**) 18FDG PET (arrowhead showing tumor) (**C**) demonstrates hypermetabolism involving only the anterior aspect of the tumor (arrowhead), whereas the 11C-Methionine PET and (**D**) depict the entirety of the tumor more conspicuously. (arrowheads outlining tumor area) Reproduced with permission from [25] © The authors 2022.

Maximizing the EOR is one of the strongest predictors of overall survival in GBM, and achieving a high EOR requires accurate visualization of tumor boundaries using both preoperative and intraoperative imaging tools [16,17]. Preoperative modalities such as MRI and PET help define tumor borders and guide surgical planning, yet all are constrained by limited spatial resolution, registration errors, and the inability to account for intraoperative changes including brain shift, tissue deformation, and cavity evolution (Figure 1) [8,17,18,19,20,21,22].

**Figure 2:**
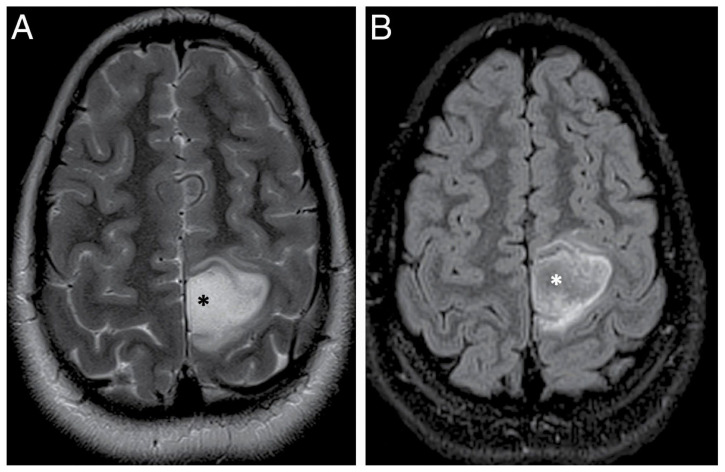
T2-FLAIR mismatch sign in an 11-year-old female patient with a diagnosis of IDH-mutant, 1p/19q non-codeleted and p53-muted anaplastic astrocytoma in the left parietal lobe. (**A**) Axial T2-weighted image demonstrating an ill-defined tumor in the left paracentral lobule region with almost homogenous hyperintense T2 signal at the center of the tumor (*) that is mostly hypointense on the corresponding FLAIR image (**B**). Reproduced with permission from: [25] © The authors 2022.

Currently, MRI is the most accessible and foundational of preoperative imaging modalities. Clinical imaging of GBM is guided by established recommendations, including the response assessment in neuro-oncology (RANO) criteria and expert consensus guidelines such as the national comprehensive cancer network which standardizes the use of structural and advanced MRI options. T1- or T2-weighted MRIs with and without gadolinium-based contrast agents are standard-of-care imaging modalities for diagnosis, surgical planning, post-treatment and surveillance in GBM [23,24]. T1-weighted gadolinium-enhanced MRI identifies areas of the blood–brain barrier (BBB) disruption typical of enhancing tumors, while T2-weighted sequences help delineate non-enhancing tumors where GBM has elements of both enhancing and non-enhancing area [8,19,20]. Fluid attenuated inversion recovery (FLAIR) MRI is a T2 technique that reduces the effect of the cerebrospinal fluid (CSF) on imaging and can enhance infiltrative regions of GBMs beyond the contrast-enhancing tumor mass. It is of note that these regions can also be mistaken for vasogenic edema without being removed or properly assessed (Figure 2) [8,19,20]. Despite these advances, MRI can only approximate the true tumor margin, and it lacks the resolution necessary to detect microscopic infiltration.

PET imaging with amino-acid tracers such as L-[methyl-^11^C] methionine (MET) and 18F fluoroethyl L tyrosine (18F FET) can improve tumor localization by highlighting metabolically active regions, particularly in lesions with ill-defined borders or extensive FLAIR abnormalities (Figure 1) [18,26]. However, PET is limited by high cost, a short tracer half-life, and inconsistent uptake, especially in non-enhancing gliomas or necrotic components of enhancing tumors [18,20,26]. Spatial resolution is also limited to the millimeter scale, which is insufficient for identifying individual migrating cells [18,26].

To address the limitations of static preoperative imaging, intraoperative techniques have become increasingly important. Intraoperative MRI and ultrasound, including modern variants such as contrast-enhanced ultrasound and neuronavigation-integrated ultrasound, help compensate for brain shift and ongoing anatomical changes during surgery [21]. Yet even these real-time modalities remain restricted by spatial resolution and cannot reliably visualize individual infiltrating GBM cells.

Fluorescence-guided surgery provides another layer of intraoperative visualization. The most widely used agent, 5-aminolevulinic acid (5-ALA), is metabolized into protoporphyrin IX and accumulates in malignant GBM cells, producing a red fluorescence under blue light without requiring BBB breakdown [27]. Despite its utility, 5-ALA lacks single-cell precision, is susceptible to photobleaching, and requires dark-field conditions [27,28].

Sodium fluorescein, which accumulates by extravasation after BBB damage, corresponds closely with contrast-enhancing margins and serves as a practical bridge between preoperative MRI and intraoperative visualization, though it risks staining edematous tissue and has limited specificity [27,29]. Indocyanine green, primarily a vascular imaging dye, offers deeper tissue penetration and low autofluorescence, but remains limited by rapid clearance, low spatial resolution, and variable utility in GBMs with heterogeneous vascular recruitment [27,30].

Across the literature, the consensus remains clear: the EOR is a major determinant of overall survival and time to recurrence, yet complete resection is fundamentally limited by the diffuse infiltration, complex vasculature, mass effect, and heterogeneous microenvironment characteristic of GBM [2,31,32]. Continued progress toward maximal safe resection will require imaging technologies capable of near-single-cell resolution to map tumor infiltration with far greater precision while preserving functionally critical tissue. Several emerging modalities with this potential are now in preclinical development and will be discussed in the following section.

## 3. High-Resolution In Vivo Imaging Modalities

### 3.1. Multiphoton Fluorescence Microscopy

**Figure 3:**
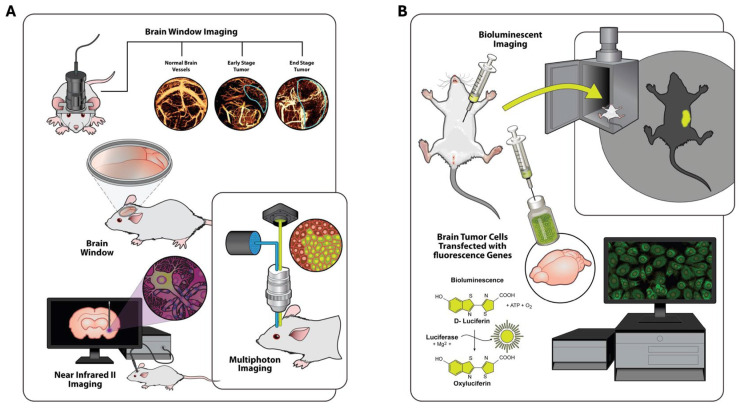
In vivo imaging techniques in rodent brain tumor models. (**A**) Brain window imaging performed with and without a microscope headpiece attachment, enabling longitudinal visualization of normal vasculature, early tumor development, and end-stage tumor progression. Multiphoton microscopy with headpiece attachment allows high-resolution fluorescent tracing of labeled tumor cells and surrounding microenvironment. Near-infrared II (NIR-II) imaging demonstrates probe insertion and fluorescent single-cell tracking in vivo. (**B**) Bioluminescent imaging of xenograft growth using luciferase-tagged human glioma cells. Following D-luciferin administration, luciferase-expressing tumor cells emit light, permitting noninvasive longitudinal monitoring and whole-body tumor cell tracking.

Multiphoton fluorescence microscopy enables deep-tissue imaging with subcellular resolution by restricting nonlinear excitation to the microscope’s focal volume, thereby reducing out-of-focus photodamage and improving penetration depth [33,34]. Within advanced optical imaging approaches, the primary multi-photon modalities used in neuro-oncology are two-photon and three-photon fluorescence microscopy (Figure 3A). While one-photon techniques such as confocal and light-sheet microscopy fall outside the true multi-photon category, they are noted here for comparison. One-photon approaches represent earlier technologies with limited penetration and a reduced ability to visualize deep tumor structures. Technological advances leading to two- and three-photon microscopy have substantially improved imaging depth, resolution, and overall capability.

Two-photon microscopy improves the limitations of one-photon approaches by using near-infrared excitation to achieve greater penetration and reduced light scattering [33,34]. Imaging performance can be further enhanced with fluorescent probes targeted to specific cellular populations. Two-photon microscopy has been extensively explored for single-cell imaging of the cortex in glioma models, but its effective depth is restricted to roughly 300–700 µm because of scattering and obstruction by dense tumor tissue (Table 1) [34]. Experimentally, two-photon microscopy has yielded invaluable insights into GBM cellular behavior, therapeutic responses, and the tumor–microenvironment (TME) interactions [34,35].

Longitudinal tracking of GBM tumors pre- and post-surgery is possible in these experimental models allowing for much of this information about tumor progression to be obtained. This is not the case in humans as longitudinal tracking using two-photon microscopy requires a craniotomy and brain window implantation. Many animal studies have utilized cranial windows with attached multi-photon microscopy hardware to monitor tumor invasion, angiogenesis, immune cell trafficking, treatment response, and TME interactions within native brain architecture (Figure 3A) [34,35,36,37,38]. One approach to bridge this gap in humans who cannot have brain windows implanted is to combine pre- and postoperative MRI for macroscopic assessment of tumor invasion with intraoperative two-photon microscopy, which correlates well with MRI findings while providing higher-resolution information on local cellular networks, the TME, and treatment biodistribution [39].

Three-photon microscopy represents the next generation of multi-photon imaging and addresses several limitations of earlier techniques. When paired with AI-based image optimization, this method, termed Deep 3P, enhances the signal-to-noise ratio during time-lapse imaging and achieves penetration depths of up to ~1.2 mm (Table 1) [34]. This extended depth enables imaging within both cortical gray matter and deeper white matter tracts in glioma animal models [34]. In combination with fluorescent reporter mouse lines, Deep 3P has allowed researchers to longitudinally monitor glioma colonization, TME interactions, and evolving vascular architecture throughout disease progression [34]. However, clinical translation remains challenging due to the need for appropriate exogenous fluorescent reporters, as current implementations rely heavily on genetically encoded fluorescence that is not directly applicable to human use. While this represents a significant barrier, the ability of Deep 3P to achieve high-resolution, deep tissue imaging highlights its potential as a powerful intraoperative tool if suitable contrast strategies can be developed.

As multi-photon methods continue to evolve toward greater depth and precision, they have also begun to interface with emerging technologies that extend imaging beyond visualization alone. One such example is Image-seq, a technique that integrates multi-photon imaging with a high-pulse-energy, low-average-power ablation laser with a micropipette to aspirate individual cells after laser-mediated tissue disruption [40]. This allows for spatial transcriptomic analysis and imaging. Initially developed in models of acute myeloblastic leukemia, Image-seq enables high-resolution imaging followed by targeted extraction of single-cell suspensions from precisely mapped regions of interest [40]. The increased spatial resolution achievable with multi-photon imaging suggests potential applicability to gliomas, particularly for guiding targeted sampling at infiltrative margins. When coupled with RNA sequencing, Image-seq could function as a single cell biopsy platform capable of identifying regions requiring future resection and inform high-precision surgical decision making. Real-time intraoperative implementation remains limited by the time required for RNA sequencing and downstream analysis. As a result, this approach is currently better suited for adjunctive or staged decision making rather than immediate surgical guidance, although advances in rapid sequencing technologies may enable future integration into intraoperative workflows.

### 3.2. Near-Infrared II Fluorescent Imaging

Near-infrared II (NIR-II) fluorescent imaging, which roughly spans the range of 1000 to 1700 nm in emission wavelengths, has emerged as a powerful optical platform for in vivo visualization of GBM. By leveraging the longer wavelength window to minimize tissue scattering, reduce autofluorescence, and improve signal-to-noise ratios relative to conventional NIR-I probes, NIR-II probes have made significant advances toward improving resolution and clinical translatability (Table 1) [41]. In preclinical orthotopic glioma and GBM models, a variety of organic and polymeric NIR-II probes have achieved high-contrast visualization of intracranial tumors that allow for the delineation of tumor margins and microvascular architecture at mesoscopic-to-cellular resolution (Figure 3A) [42,43,44]. These optical properties enable deeper tissue penetration and improved visualization of intracranial structures, increasing the chance of translatability with a high degree of resolution at GBM tumor margins.

Semiconducting polymer-based NIR-II nanoprobes have recently been used both for high-contrast transcranial in vivo imaging of orthotopic GBM as well as NIR-II guided photothermal ablation [41]. The use of NIR-II for imaging and photothermal ablation has also been used in a biodegradable nanoprobe technique with higher photothermal efficiency [44]. The biodegradable nature and systemic clearance of this nanoprobe is a necessary innovation since this approach has been previously limited by poor inorganic cytocompatibility [44]. These developments show the potential of NIR-II platforms to combine high-resolution tumor visualization with targeted therapeutic intervention. While further validation of biodegradation, biocompatibility, and photothermal safety is required in humans, this approach represents a promising strategy for integrating high-resolution imaging and therapy in GBM management.

Achieving this integration in vivo requires effective tumor-specific targeting and delivery strategies to ensure sufficient probe accumulation within GBM tissue. One such approach utilizes a biomimetic NIR-II platform consisting of semiconducting polymer dots (Pdots) coated with homologous C6 glioma cell membranes [42]. This biomimetic coating enhances tumor recognition and uptake, resulting in strong emissions at around 1055 nm in the NIR-II window and improved intratumoral accumulation compared to the uncoated Pdots, thereby enhancing tumor visualization [42]. Approaches aimed at improving delivery across the BBB have also been explored, including aggregation-induced emission based NIR-II nanotheranostics capable of crossing disrupted BBBs and providing high-resolution tumor contrast [43]. Through this approach, more effective targeting of photothermal therapy in orthotopic GBM models was possible [43]. Together, these advances demonstrate that optimizing targeting and delivery is a critical step toward translating NIR-II theranostic platforms into clinical viable tools.

### 3.3. Bioluminescent Imaging

Bioluminescent imaging (BLI) is an optical technique used exclusively in preclinical models because it requires genetic modification of tumor or therapeutic cells to express luciferase enzymes, most commonly firefly luciferase (Figure 3B). Despite this limitation, BLI has become a widely used tool in glioma research because emitted light intensity correlates directly with viable cell number, allowing highly sensitive, noninvasive, and longitudinal monitoring with minimal physiological disruption [9,10,45,46]. Unlike fluorescence-based approaches, BLI does not rely on external excitation; instead, light is produced intrinsically through the luciferase–luciferin reaction and can be detected transdermally with high signal-to-noise ratios (Table 1) [9,10,46].

In GBM animal models, luciferase-expressing tumor cells are typically generated in vitro and then implanted heterotopically, as the skull severely attenuates bioluminescent photons and prevents reliable intracranial signal detection [9,10,45]. BLI in its current state, as an in vivo imaging system used to track tumor growth, cellular recruitment, or therapy delivery, is unsuitable for clinical translation, since reliable signal capture would require frequent craniotomies. Additional challenges include immune responses to luciferase expression reported in some GBM models [46] and instability of reporter expression in certain cell populations, where transgene silencing or decreasing expression over time can falsely suggest reduced cell survival rather than a simple loss of signal [45].

Although BLI currently remains a preclinical modality, its use in tracking stem cell-based or T-cell-based therapies represents one of the most plausible avenues for eventual human adaptation, contingent on advances that ensure the safety of luciferase-expressing cell populations. Animal models have shown that mesenchymal stem cells can be transduced with luciferase and, in some systems, co-labeled with quantum dots optimized for NIR intravital microscopy, enabling precise monitoring of cell delivery and distribution [47]. These strategies allow real-time visualization of therapeutic cell localization, migration, and persistence in vivo and can be paired with anatomic imaging modalities such as T2-weighted MRI to concurrently assess changes in tumor burden [47,48].

### 3.4. Photoacoustic Imaging

Photoacoustic imaging (PAI) translates pulsed near-infrared light into ultra-high-frequency sound (ultrasound), enabling deeper tissue visualization than conventional optical modalities (Table 1) [49,50,51]. In heterotopic GBM models, PAI has been utilized for noninvasive assessment of tumor burden and for the evaluation of nanoparticle biodistribution in intact skulls [49]. Nanomaterial-based platforms such as π-conjugated polymer nanoparticles, semiconducting absorbers, and NIR-responsive organic probes improve PAI further due to their excellent optical absorption capacity, strong photothermal conversion efficiency, and optimized surface chemistry for the traversal of the BBB or specific uptake in tumors [43,51]. In previous experiments, these agents generated stable photoacoustic signals under repeated pulsated-laser excitation and supported combined imaging and therapy. This allowed for real-time monitoring of biodistribution and treatment response in glioma-bearing mice [50]. These properties position PAI as a unique modality capable of combining optical contrast with ultrasound penetration, enabling deeper, high-contrast imaging of intracranial tumors compared to purely optical techniques.

Multispectral PAI allows for functional assessment of parameters like oxygen saturation, hemoglobin concentration, and vascular density, which are vital markers of hypoxia, angiogenesis, and vascular remodeling secondary to treatment in GBM [51]. While PAI has progressed to be a high-contrast and intermediate-depth imaging tool that can assess the structural and functional properties of GBM, it is currently still limited in resolution without precise markers in GBM cells. Some of the limitations to overcome before translation to humans include further enhancing spatial resolution compared to microscopy-based techniques, finding appropriate exogenous contrast agents, and distinguishing the tumor from surrounding tissue without targeted probes. In clinical practice, PAI is most likely to serve as a complementary intraoperative or perioperative imaging modality that can give greater depth penetration and vascular characteristics compared to other techniques.

### 3.5. Optical Coherence Tomography

**Figure 4:**
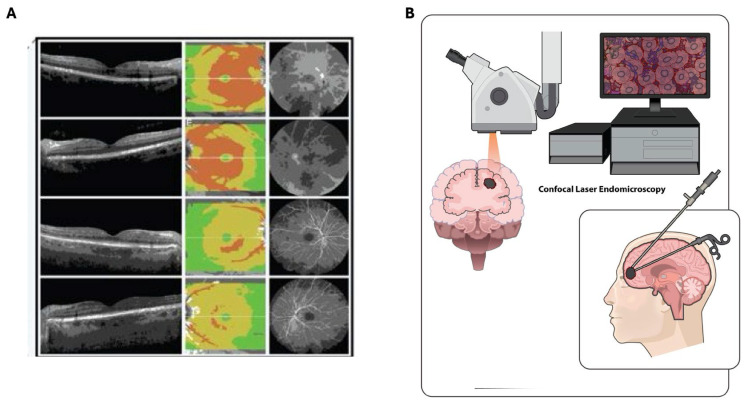
Clinically used high-resolution in vivo imaging modalities. (**A**) Optical coherence tomography (OCT) enabling noninvasive, cross-sectional visualization of cellular and microstructural layers. (**B**) Confocal laser endomicroscopy (CLE) demonstrating the use of confocal probes or objectives inserted into the brain to provide real-time, high-resolution cellular imaging during neurosurgical resection procedures.

Optical coherence tomography (OCT) is a noninvasive imaging technique that reconstructs tissue structure by analyzing backscattered optical signals without the need for contrast agents or fluorescent dyes (Figure 4A) [52,53]. OCT provides micrometer-scale resolution (approximately 10–20 μm) with a penetration depth of roughly 1–2 mm, which corresponds closely with the depth of regions that experience cancer infiltration during surgical resection (Table 1) [54,55]. Advances in Fourier domain technology now allow three-dimensional, label-free volumetric in vivo imaging, which has been utilized in fields such as ophthalmology, dermatology, and cardiology [53].

OCT signal-derived parameters provide insight into tissue composition and microstructure [54,56]. Optical attenuation coefficients reflect intrinsic scattering properties influenced by tissue architecture, including myelinated fibers and cellular density [54,56]. For example, highly scattering myelin-rich white matter produces distinct signal characteristics compared to GBM which has increased cellularity, vascularization, and myelin degradation [54]. These differences can be leveraged to generate color-coded attenuation maps, which are a major focus of OCT research for tissue differentiation and surgical guidance [56]. Additional contrast mechanisms, including interchannel attenuation and refined speckle contrast, further enhance sensitivity to microstructural heterogeneity and improve discrimination between malignant and non-malignant tissue, and are particularly valuable when combined in multiparametric analyses or without an accurate attenuation map [54,56,57].

In addition to attenuation-based approaches, advanced analytical and functional extensions of OCT provide further discriminatory capability. Texture-based analyses, such as homogeneity and variance from gray-level co-occurrence matrices, offer quantitative measures of tissue heterogeneity that improve classification of tumor versus normal tissue [52]. OCT angiography extends these capabilities by enabling quantitative assessment of tumor-associated vascular changes, including vessel density, diameter, and fractal complexity during tumor progression [52]. Studies have also explored OCT-based analysis of tumor-related macrophages, contrast-enhanced Doppler methods for visualizing capillary networks, and optical coherence microscopy for tracking GBM development in animal models but without translation to humans [52].

Several limitations account for reduced clinical translation to larger patient populations such as challenges in acquisition speed, processing requirements, and signal attenuation. One limitation in attenuation mapping is that they are derived from ex vivo tissue samples, which may reduce their applicability in real-time in vivo settings because of tissue property differences, signal conditions, and limitations with algorithms in living brains [54,56]. Computational approaches are also a critical barrier to implementation because traditional approaches that estimate attenuation coefficients improve signal stability but reduce spatial resolution and newer approaches that preserve spatial detail require more advanced processing [55]. Other limitations include small sample sizes of both ex vivo attenuation mapping studies and of in vivo trials, variability in attenuation trends, and the need for supervised image analysis from tissue heterogeneity and signal variability which impact surgical timing, training, and accuracy [54]. The continued development of automated, real-time analysis methods will be essential for integrating OCT into surgical workflows for the accurate delineation of GBM margins.

### 3.6. Confocal Laser Endomicroscopy

Confocal laser endomicroscopy (CLE) enables real-time, high-resolution cellular imaging directly in the surgical field, offering “optical biopsy” capability by visualizing tumor tissue, infiltrative margins, and microvascular architecture without the delays of frozen-section histology (Figure 4B) [27,28,58]. This enables intraoperative decision making at a cellular level, potentially reducing reliance on time-consuming histopathological workflows (Table 1).

In in vivo human brain tumor applications, CLE has achieved diagnostic accuracies of 90%, with sensitivity and specificity approaching those of conventional histology, making it especially valuable for identifying infiltrative glioma margins [27,58]. Fluorescent contrast agents or autofluorescence can also be paired with CLE and have the potential to assist surgeons in delineating the true extent of tumor tissue during resection of GBM by providing immediate feedback on tissue status [58].

CLE is restricted from widespread adoption in neurosurgical practice due to several limitations. First this technique relies on a limited field of view and shallow imaging depth which can cause incomplete assessment of heterogeneous tumor margins. Image misinterpretation can also be challenging, and specialized training and experience are required to distinguish tumors from non-tumor tissue as well as significant time to orient to the field of view. These things can impact the efficacy of exogenous fluorescent agents with prolonged or incorrect exposure times. Intraoperative factors such as motion artifacts, blood contamination, and difficulty maintaining optimal probe positioning can further complicate image acquisition. CLE has significant clinical value for highly trained surgeons with experience in the identification of tumor margins, where it can act as a confirmatory tool for maximal resection; however, its utility may be limited for less experienced users.

### 3.7. Raman Spectroscopy

Label-free optical imaging utilizes natural properties of samples such as the refractive index variations, autofluorescence, molecular vibrations, birefringence, scattering, and absorption properties to generate image contrast without dyes or labels. These techniques depend on a specific source of intrinsic contrast, which requires adjustments to the optical setup [53]. One such label-free technique is Raman spectroscopy which detects inelastic scattering from molecular vibrations and provides biochemical information about tissue composition [53,59,60]. Raman-based approaches have been shown to differentiate GBM tissue from normal brain based on lipid, protein, and water content, as well as specific spectral peaks associated with tumor metabolism and cellular composition [61,62,63].

Traditional (linear) Raman imaging is not well suited for in vivo applications due to weak scattering signals, as only a small fraction of incident photons undergo inelastic scattering, resulting in low signal-to-noise ratios and long acquisition times [59,61]. These limitations also restrict spatial sampling, as spectra are often acquired from single points rather than large tissue regions [61].

To overcome these limitations, nonlinear Raman techniques such as coherent anti-Stokes Raman scattering (CARS) and stimulated Raman scattering (SRS), were developed to enhance signal intensity and suppress background autofluorescence [53]. Two-channel SRS imaging targeting lipid and protein vibrations allows differentiation between myelin-rich white matter and protein-dense tumor regions and has been used to identify tumor margins not visible under standard surgical conditions [61].

Building on these advances, Raman spectroscopy has been adapted for intraoperative use through the development of handheld fiber-optic probes, enabling rapid, point-based spectral acquisition directly from tissue surfaces. These probes can acquire spectra in approximately 0.2 s with a sampling depth of 1 mm and have demonstrated the ability to distinguish tumor from normal brain tissue with high sensitivity and specificity in human patients (Table 1) [63]. Raman has also been shown to detect infiltrative tumor cells beyond radiographically abnormal regions, highlighting their potential to improve surgical margin identification [61,62]. These techniques are also further enhanced by computational analysis advances such as machine learning and neural networks that improve the robustness of Raman signal interpretation even in the presence of environmental interference [64,65].

A significant limitation of these, and most optical imaging, is the depth limitation which is due to a limited illumination-source-to-detector separation [60,66]. This requires physical contact or near-contact of the probe with the surgical cavity wall to validate margin status.

### 3.8. Autofluorescence Microscopy

Autofluorescence microscopy (aFM) measures the natural fluorescence of biomolecules like NAD(P)H, FAD, and tryptophan, which reflect the metabolic activity of cells [53]. These endogenous fluorophores can be excited by single-photon or multi-photon interactions, with multi-photon excitation offering higher resolution, an improved signal-to-noise ratio, deeper tissue penetration, and optical sectioning (Table 1). This label-free approach allows real-time tissue assessment without the need for exogenous contrast agents, avoiding issues related to dye administration, uptake, and clearance [67].

To evaluate the in vivo applicability of autofluorescence imaging, preclinical studies have investigated its ability to distinguish tumor and non-tumor tissue based on metabolic differences. Chang et al. developed an optical fiber-embedded needle probe to measure the autofluorescence of both cerebral hemispheres in a GBM rat model at various depths 5 days after the implantation of C6 glioma cells and primary mixed glial cells (PGCs) [68]. Comparative analyses of corresponding areas by histopathology and autofluorescence revealed significant differences among the normal tissue, infiltration zone, tumors, and the contralateral cerebral hemispheres [68].

Building on these preclinical findings, autofluorescence imaging has also been applied in human brain tissue using CLE systems. The integration of CLE with autofluorescence allows for the visualization of cellular and structural features such as cytoplasmic fluorescence, elastin fibers, blood vessels, and punctate or diffuse fluorescence patterns [67]. These distinct patterns have been observed between tumor and non-tumor tissue with tumors demonstrating more diffuse and structurally heterogeneous fluorescence while non-tumor tissue exhibits higher densities of autofluorescent cells [67].

Several limitations to autofluorescence imaging remain such as weaker signals than exogenous fluorescence, requiring higher detector sensitivity and increased image noise. Intraoperative imaging without fluorescent markers is further complicated by motion artifacts, blood contamination, and focal plane focusing [67]. These factors highlight the need for significant optimization of autofluorescence before it is readily adopted in clinical settings to distinguish tumor margins.

### 3.9. Fluorescence Macroscopy

Fluorescence macroscopy and time-resolved fluorescence techniques provide label-free, wide-field and depth-sensitive approaches for intraoperative tissue characterization (Table 1). Fluorescence lifetime imaging enables rapid, non-contact assessment of endogenous fluorophores with each point measurement acquired in less than 1 μs, allowing free-hand scanning of large tissue areas during surgery [69]. Unlike contact-based optical methods, fluorescence lifetime imaging can operate under ambient light conditions and be integrated into the neurosurgical workflow, a new advancement that pushes this technique beyond early in vitro or ex vivo validation studies [69]. Early clinical studies demonstrate that fluorescence lifetime signatures vary with tumor cellularity and prior treatment history, suggesting potential utility for identifying infiltrative tumor regions [69].

Fluorescence lifetime imaging microscopy (FLIM) further extends these capabilities by providing high-resolution, fiber-based imaging of tissue autofluorescence in the surgical field. Intraoperative FLIM systems have demonstrated the ability to distinguish GBM from normal cortex based on differences in intensity and lifetime, with tumor tissue exhibiting longer fluorescence lifetimes despite reduced signal intensity [70]. Importantly, fluorescence lifetime contrast is relatively robust to variation in tissue surface irregularity, blood presence, and illumination conditions, making it an ideal candidate for intraoperative use with advantages over Raman spectroscopy.

Time resolve laser induced fluorescence spectroscopy (TR-LIFS) provides complementary spectral and temporal information by analyzing fluorescence decay profiles across multiple wavelength bands. It is also widely used to study radiation therapy and tumor size but requires histological samples to be taken for imaging so it is largely excluded from this discussion. In vivo studies utilizing TR-LIFS have demonstrated that it can differentiate normal cortex, white matter, and glioma tissue based on distinct emission peaks and fluorescence lifetimes, including the loss or reduction in specific emission features in tumor tissue [71]. While these techniques show potential for near-real-time classification, variability in GBM fluorescence and differences between ex vivo and in vivo measurement highlight the need for further validation with larger cohort studies before it is adopted into use.

## 4. Barriers to Single-Cell Fluorescent Labeling

### 4.1. Fundamental Signal Limitations and Amplification Strategies at the Single-Cell Scale

Achieving reliable single-cell resolution with fluorescent reporters in vivo remains fundamentally constrained by limitations in molecular abundance, signal generation, intracellular target accessibility, and delivery across the BBB [72,73]. Given these combined constraints, no single probe or reporter class is universally optimal for GBM imaging. Therefore, currently effective imaging strategies must accept a fundamental trade-off between imaging depth, spatial resolution, and procedural invasiveness.

Molecular abundance is one of the most critical barriers, as individual cells express a finite number of target molecules. Many clinically relevant biomarkers, including low-copy-number proteins and transcripts, provide limited opportunities for probe binding and subsequent signal generation [74,75,76,77]. As a result, feasibility must be grounded in realistic estimates of target copy number per cell. The detectable signal reflects the cumulative efficiency of a multistep cascade, including target availability, probe–target interaction, photon emission, and detector capture, with signal loss occurring at each stage [77,78,79]. These constraints impose a fundamental trade-off between the detectable signal and biologically relevant target selection, as low-abundance markers that best reflect tumor biology are often the most difficult to visualize in vivo.

To address these constraints, several signal amplification strategies have been developed, broadly categorized as optical, enzymatic, and genetic approaches [77,78]. Optical amplification methods, such as quantum dots and fluorescent nanoprobes, increase photon output per binding event and offer advantages including photostability, size-tunable emission, and resistance to photobleaching [80,81,82,83]. These platforms have enabled in vivo labeling of GBM cells and infiltrative tumor margins, as demonstrated with GFAP-targeted CdTe quantum dots [84]. However, their effectiveness remains limited by probe-to-target stoichiometry and the availability of accessible binding sites, preventing compensation for low-abundance or poorly accessible targets [80,81,82,83].

Enzymatic amplification strategies can produce substantial signal enhancement, as seen in assays such as ELISA and Western blotting, but are inherently incompatible with real-time in vivo imaging due to their reliance on ex vivo processing [77,79]. Genetic amplification offers an alternative by enabling cells to generate a signal intrinsically through reporter gene expression. Systems based on luciferase, NIR fluorescent proteins, and multimodal constructs such as ferritin-EGFP fusions and lentiviral multi-reporter platforms have enabled longitudinal and multimodal imaging in GBM models [77,78,79,85,86,87,88,89,90,91,92]. However, these approaches face significant limitations related to delivery efficiency, the heterogeneity of expression, and challenges in clinical translatability [77,79,86]. In addition, the requirement for stable gene delivery introduces ethical and safety considerations that are not present with exogenous contrast agents.

Despite the diversity of amplification strategies, all approaches remain constrained by the same underlying limitations in target availability, delivery, and signal generation. Consequently, imaging of low-copy targets at single-cell resolution in vivo remains challenging, and further advances must be guided by the specific biological objective rather than the capabilities of any individual modality. This often requires prioritizing accessible or high-abundance targets over those most reflective of disease state, a significant limitation with extremely variable cells such as GBM.

### 4.2. Accessibility of Targets Within the Cell

Subcellular localization represents a significant barrier to in vivo molecular imaging, as many of the most biologically informative targets in GBM reside within intracellular compartments that are inaccessible to conventional probes. Critical oncogenic pathways and mediators of therapeutic resistance are often localized to the cytoplasm or nucleus, where they are shielded by the plasma membrane and the nuclear envelope, limiting direct probe access [74,75,76]. Because most exogenous agents, including antibodies and larger nanoparticles, cannot readily penetrate intact cellular membranes, imaging strategies are inherently biased toward surface-expressed markers. However, these markers may not accurately reflect underlying tumor biology, particularly for processes such as proliferation, DNA damage response, and therapeutic resistance, creating a fundamental limitation in marker selection [74,75].

To address this constraint, alternative strategies have been developed to enable intracellular signal generation. Reporter-based systems, including genetically encoded constructs delivered via viral vectors, allow cells to produce detectable signals from within, enabling longitudinal imaging of cellular dynamics in GBM models [15,93,94]. Approaches based on CRISPR-Cas9 and Cre-Lox systems provide pathway-specific and conditional control of reporter expression, while fluorescent probes targeting DNA damage (e.g., PARP-based systems) and enhanced luciferase platforms improve sensitivity and signal output [15,93,94,95,96,97,98,99]. Collectively, these methods demonstrate that intracellular processes can be visualized under controlled conditions in preclinical models.

One large limitation of these promising experiments is the requirement for genomic manipulation and/or viral transduction. These manipulations introduce challenges related to delivery efficiency, expression heterogeneity or failure, and potential insertional effects [77,79,86]. Even when successful, signal generation is constrained by binding kinetics, competition with endogenous ligands, and saturation effects, which limit quantitative accuracy at the single-cell level [74,78]. These constraints impose practical limits on both sensitivity and reproducibility in vivo.

As a result, intracellular targeting strategies face a critical trade-off between biological specificity and clinical feasibility. While intracellular markers provide more direct insight into tumor biology, the methods required to access them are not readily translatable to human use. Consequently, intracellular accessibility remains a major barrier and potentially necessitates the use of approaches that integrate complementary marker classes to improve detection of residual GBM cells.

### 4.3. Delivery Barriers Unique to the Brain

The BBB is a unique feature of the brain that has long complicated both molecular imaging and therapeutic delivery, as it restricts the systemic transport of probes into brain tissue [72,73,100,101]. Although the BBB is frequently compromised within the tumor core of GBM, this disruption is highly heterogeneous and spatially variable, resulting in uneven probe accumulation and reduced sensitivity for detecting infiltrative tumor margins [102,103,104]. As a result, probes that perform well in enhancing tumor regions often fail to identify dispersed tumor cells at the invasive edge, creating a limitation in both marker selection and detection sensitivity.

Efforts to overcome BBB-mediated exclusion have focused on engineered nanoparticle systems designed to enhance transport and tumor-specific accumulation. For example, angiopep-2-conjugated superparamagnetic iron oxide nanoparticles (SPIONs) exploit receptor-mediated transcytosis to cross the BBB and improve tumor delineation [105]. Similarly, targeted and surface-modified nanoparticles, including TSPO-targeted and PEG-based platforms, have demonstrated improved tumor specificity and the ability to reveal vascular and infiltrative features not detected by conventional contrast agents [102,103,104,106,107,108]. These advances are promising for future drug delivery and research is constantly happening to identify new drug carriers that can cross the BBB.

Even when the BBB is bypassed, probe distribution within brain tissue remains constrained by the physical properties of the tumor microenvironment. Dense extracellular matrix architecture and elevated interstitial fluid pressure generate outward convective forces that limit penetration into infiltrative regions, further reducing detection sensitivity [102,104]. Direct intratumoral delivery intraoperatively can achieve highly localized labeling, but this approach is spatially and temporally restricted and not scalable for comprehensive whole-brain imaging assessment [84]. Consequently, no single fluorescent marker delivery strategy is yet sufficient for comprehensive imaging.

## 5. Implications for Detection of Residual GBM Cells

Single-cell imaging enables mapping of tumor heterogeneity and tracking of infiltrative GBM cells, which are critical for identifying therapy-resistant niches and the cellular populations responsible for recurrence [34,35,109,110]. The high degree of drug resistance and recurrence in GBM is driven in large part by profound cellular and genetic heterogeneity within the TME, allowing for the survival of resistant subpopulations, particularly GSCs which evade conventional treatment and contribute to tumor regrowth [111,112,113]. Residual tumor cells are often spatially dispersed beyond radiographically defined tumor margins and exist in low-density, infiltrative states that are not detectable using conventional imaging [11]. These cells also exhibit diverse phenotypic and metabolic profiles that further complicate detection [2].

Molecular approaches such as single-cell RNA sequencing (scRNA-seq) and spatial transcriptomics have provided important insight into this heterogeneity and are useful in identifying potentially unique GBM markers for tracing or probe development. GBM markers are difficult to make because of the diversity of malignant cell states, including previously uncharacterized subpopulations, particularly in infiltrative regions where marker expression is variable and often reduced [114]. As a result, the identification of robust, universally applicable imaging targets remains a major challenge, directly impacting the ability to detect residual tumor cells in vivo.

These biological constraints have direct consequences for how residual tumor detection must be approached. First, no single molecular marker is sufficient for identifying all malignant cell populations, particularly at infiltrative margins. Second, the low-density distribution of residual cells imposes strict requirements on spatial resolution and signal sensitivity, as even small decreases in detection efficiency may result in clinically meaningful tumor burden being missed. Third, because many of the most biologically relevant processes contend with limited accessibility and signal generation, there are further constraints to achieving sensitivity in vivo.

These constraints suggest that effective detection of residual GBM will require a shift from modality-centered approaches toward detection strategies that are explicitly designed around tumor biology. In practice, this may involve the use of multiple complementary targets to account for cellular heterogeneity, the prioritization of markers that remain expressed in infiltrative regions, and the integration of imaging modalities that balance spatial resolution with depth and coverage. Rather than identifying a single optimal technique, the goal becomes minimizing false-negative detection across diverse tumor states and microenvironments.

From a clinical perspective, this reframes the role of in vivo optical imaging. Instead of serving as standalone tools for complete tumor visualization, these technologies may be most effective when used to interrogate high-risk regions, such as infiltrative margins or areas of suspected residual disease, where conventional imaging lacks sensitivity. In this context, high-resolution optical methods can provide localized, cell-level validation of tumor presence, complementing broader imaging modalities that define overall tumor extent. Ultimately, improving detection of residual GBM cells will depend on aligning imaging strategies with the biological realities of tumor heterogeneity, rather than relying solely on advances in resolution or contrast.

## 6. Future Directions and Challenges

Bringing single-cell in vivo imaging into routine surgical practice will require substantial technological and regulatory progress, as many current techniques carry unacceptable risks, impose impractical longitudinal monitoring requirements, or rely on labeling agents that lack FDA approval. A primary barrier to translation is the performance of these modalities in human brain tissue, which is larger and demands far greater imaging depth. Human brain tissue also causes increased light scattering and presents a reduced field of view. All of this complicates reliable margin assessment. Emerging innovations such as Deep3P, Image-seq, and AI-driven image processing attempt to address some of these limitations, but substantial challenges remain.

Beyond the modality-specific constraints described earlier, significant barriers exist in cost, workflow integration, safety, and equipment operation. High acquisition and maintenance costs limit both patient access and hospital adoption. Intraoperative imaging adds time to surgical procedures, as probe placement, repositioning, and image acquisition are often slow. Prolonged operative times not only increase anesthetic risk, but also reduce surgical throughput, placing additional strain on neurosurgeons and operating room scheduling.

Another critical obstacle is the FDA approval and safety profile of contrast agents, fluorescent markers, and other labeling strategies. Achieving true single-cell discrimination requires markers with exceptional specificity and brightness, yet most candidates remain experimental and lack established safety in humans. Furthermore, while maximizing the EOR is essential, preserving functional healthy tissue is equally vital. Thus, any imaging approach must avoid compromising healthy brain regions for the sake of deeper penetration or probe insertion. The future utilization of these techniques will rely on finding a balance between pursuing microscopic tumor projections and tissue sparing.

Lastly, successful clinical deployment of advanced in vivo imaging will depend on the physical footprint and flexibility of the equipment. Many systems are large, rigid, and difficult to maneuver, complicating surgical workflows and posing potential hazards if rapid intervention is required during patient instability. Meaningful clinical translation will require imaging platforms that are more compact, mobile, and able to be seamlessly integrated into existing neurosurgical environments.

## 7. Conclusions

Single-cell in vivo imaging marks a paradigm shift in how we understand and manage GBM. These technologies enable dynamic visualization of tumor and immune cell behavior at unprecedented resolution. While barriers to clinical translation remain, ongoing innovation in imaging physics, molecular labeling, and computational analysis will pave the way for precision neuro-oncology guided by real-time, cellular-level insights.

## Figures and Tables

**Table 1:** Comparative summary of in vivo optical modalities.

Modality	Resolution	Penetration Depth	Advantages	Limitations	Clinical Status	Key Barriers to Clinical Implementation	Primary Biological/Clinical Applications
Multiphoton Microscopy (2P/3P)	Subcellular (~µm)	300–700 µm (2P); ~1.2 mm (3P)	High-resolution; real-time; TME dynamics	Limited depth; fluorescent reporters	Preclinical	Reporter dependence; limited field of view	Tumor heterogeneity mapping; TME interactions; vascular dynamics
Near-Infrared II (NIR-II) Fluorescence Imaging	Mesoscopic to near cellular	Several mm	Deep penetration; low scattering; theranostic	BBB delivery; probe safety	Preclinical/Early translational	Probe insertion; regulatory approval; targeting specificity	Tumor margin delineation; drug delivery tracking; photothermal therapy guidance
Bioluminescence Imaging (BLI)	Low spatial resolution	Deep in heterotopic tumors (limited by skull)	High sensitivity; no excitation light; longitudinal tracking	Requires genetic modification; poor spatial resolution	Preclinical	Need for reporter genes; low spatial resolution; not clinically translatable	Tumor burden monitoring; longitudinal tracking; therapy response in models
Photoacoustic Imaging (PAI)	~100 µm	mm–cm	Optical contrast + ultrasound depth; functional imaging	Lower resolution; contrast dependence	Emerging/Early clinical	Need for targeted contrast agents; resolution limitations; system integration	Vascular imaging; oxygenation/hypoxia mapping; treatment response monitoring
Optical Coherence Tomography (OCT)	~10–20 µm	1–2 mm	Label-free; real-time; microstructure	Limited depth; computational complexity	Early clinical	Limited penetration; interpretation variability; workflow integration	Tumor vs normal tissue differentiation; margin assessment; microstructural mapping
Confocal Laser Endomicroscopy (CLE)	Cellular (~µm)	0.5–1 mm	Optical biopsy; high accuracy	Small FOV; susceptible to artifact	Clinically available (limited)	Operator dependence; limited coverage; contrast variability	Intraoperative tumor identification; margin validation; histology-like imaging
Raman Spectroscopy (CARS/SRS)	Cellular (~µm)	~1 mm	Biochemical specificity; label-free	Weak signal; shallow depth	Early clinical	Signal sensitivity; acquisition time; probe design	Tumor margin detection; metabolic profiling; molecular composition analysis
Autofluorescence Microscopy (aFM)	Cellular–subcellular	~mm	Label-free; metabolic contrast	Weak signal; noise	Preclinical/Early clinical	Low specificity; motion artifacts; signal variability	Metabolic state assessment; tumor vs normal differentiation
Fluorescence Macroscopy (FLIM/TR-LIFS)	Cellular (~µm)	Surface to few mm	Rapid; wide-field; non-contact	Ex vivo to in vivo variability	Early clinical/Investigational	Limited specificity; calibration; validation	Wide-field tumor detection; metabolic imaging; intraoperative guidance

## Data Availability

No new data were created or analyzed in this study. Data sharing is not applicable to this article.

## Abbreviations

The following abbreviations are used in this manuscript:

GBM	glioblastoma multiforme
MRI	magnetic resonance imaging
GSCs	glioma stem like cells
EOR	extent of resection
PET	positron emission tomography
BBB	blood–brain barrier
FLAIR	fluid attenuated inversion recovery
CSF	cerebrospinal fluid
MET	methionine
FET	fluoroethyl L tyrosine
5-ALA	5 aminolevulinic acid
TME	tumor microenvironment
NIR-II	near-infrared II
Pdots	polymer dots
BLI	bioluminescent imaging
PAI	photoacoustic imaging
OCT	optical coherence tomography
CLE	confocal laser endomicroscopy
CARS	coherent anti-Stokes Raman scattering
SRS	stimulated Raman scattering
aFM	autofluorescence microscopy
PGCs	primary mixed glial cells
PARPi-FL	PARP targeted fluorescent probes
SPIONs	superparamagnetic iron oxide nanoparticles
LRP-1	lipoprotein receptor related protein
scRNA-seq	single-cell RNA sequencing
ST	spatial transcriptomics
ART	adaptive radiotherapy
ICI	immune checkpoint inhibitors
IONPs	ultrasmall iron oxide nanoparticles
NP	nanoparticles
